# Real-world assessment: effectiveness and safety of extended-release calcifediol and other vitamin D therapies for secondary hyperparathyroidism in CKD patients

**DOI:** 10.1186/s12882-022-02993-3

**Published:** 2022-11-11

**Authors:** Michael J. Germain, Subir K. Paul, George Fadda, Varshasb Broumand, Andy Nguyen, November H. McGarvey, Matthew D. Gitlin, Charles W. Bishop, Philipp Csomor, Stephen Strugnell, Akhtar Ashfaq

**Affiliations:** 1 Renal Transplant Associates of New England, Springfield, MA USA; 2 Shoals Kidney and Hypertension Center, Florence, AL USA; 3 California Institute of Renal Research, San Diego, CA USA; 4 South Texas Renal Care Group, San Antonio, TX USA; 5 BluePath Solutions, Los Angeles, CA USA; 6 OPKO Health, Inc, 4400 Biscayne Blvd, Miami, FL 33137 USA; 7 CSL-Vifor Pharma, Glattbrugg, Switzerland

**Keywords:** Extended-release calcifediol, Real-world, Secondary hyperparathyroidism, Vitamin D insufficiency, Vitamin D therapy

## Abstract

**Introduction:**

Extended-release calcifediol (ERC), active vitamin D hormones and analogs (AVD) and nutritional vitamin D (NVD) are commonly used therapies for treating secondary hyperparathyroidism (SHPT) in adults with stage 3–4 chronic kidney disease (CKD) and vitamin D insufficiency (VDI). Their effectiveness for increasing serum total 25-hydroxyvitamin D (25D) and reducing elevated plasma parathyroid hormone (PTH), the latter of which is associated with increased morbidity and mortality, has varied across controlled clinical trials. This study aimed to assess real-world experience of ERC and other vitamin D therapies in reducing PTH and increasing 25D.

**Methods:**

Medical records of 376 adult patients with stage 3–4 CKD and a history of SHPT and VDI from 15 United States (US) nephrology clinics were reviewed for up to 1 year pre- and post-ERC, NVD or AVD initiation. Key study variables included patient demographics, concomitant usage of medications and laboratory data. The mean age of the study population was 69.5 years, with gender and racial distributions representative of the US CKD population. Enrolled patients were grouped by treatment into three cohorts: ERC (*n* = 174), AVD (*n* = 55) and NVD (*n* = 147), and mean baseline levels were similar for serum 25D (18.8–23.5 ng/mL), calcium (Ca: 9.1–9.3 mg/dL), phosphorus (P: 3.7–3.8 mg/dL) and estimated glomerular filtration rate (eGFR: 30.3–35.7 mL/min/1.73m^2^). Mean baseline PTH was 181.4 pg/mL for the ERC cohort versus 156.9 for the AVD cohort and 134.8 pg/mL (*p* < 0.001) for the NVD cohort. Mean follow-up during treatment ranged from 20.0 to 28.8 weeks.

**Results:**

Serum 25D rose in all cohorts (*p* < 0.001) during treatment. ERC yielded the highest increase (*p* < 0.001) of 23.7 ± 1.6 ng/mL versus 9.7 ± 1.5 and 5.5 ± 1.3 ng/mL for NVD and AVD, respectively. PTH declined with ERC treatment by 34.1 ± 6.6 pg/mL (*p* < 0.001) but remained unchanged in the other two cohorts. Serum Ca increased 0.2 ± 0.1 pg/mL (*p* < 0.001) with AVD but remained otherwise stable. Serum alkaline phosphatase remained unchanged.

**Conclusions:**

Real-world clinical effectiveness and safety varied across the therapies under investigation, but only ERC effectively raised mean 25D (to well above 30 ng/mL) and reduced mean PTH levels without causing hypercalcemia.

## Background

Chronic kidney disease (CKD) is a growing global health concern, projected to be one of the top four leading causes of potential years of life lost by 2040. The many complications of CKD can include secondary hyperparathyroidism (SHPT) and vitamin D insufficiency (VDI) [1]. As kidney function deteriorates, significant alterations develop in the metabolism of calcium (Ca), phosphorus (P) and vitamin D which cause increased production and secretion of parathyroid hormone (PTH). This combination of decreased kidney function, mineral abnormalities and high rates of comorbidities results in a reduced quality of life for many patients [2, 3]. SHPT frequently develops as a consequence of abnormalities in these biochemical parameters and low levels of both serum total 25-hydroxyvitamin D (25D) and 1,25-dihydroxyvitamin D (1,25D) play a major role in its progression [4]. The ensuing parathyroid hyperplasia and overproduction of PTH can result in mineral and bone metabolism imbalances [5–7] that lead to loss of bone mineral density and increased risk of bone fractures [6].

Concurrent diagnoses of CKD and SHPT have been linked to increased risk of kidney disease progression, cardiovascular disease, and death [8–12]. Patients with CKD and SHPT report significantly higher medical costs and healthcare resource utilization than those who have CKD alone [9, 11, 13]. Poor vitamin D status and high PTH levels usually develop in patients who have stage 3 to 5 CKD, and they can emerge as soon as stage 2. Early and sustained control of SHPT is necessary to bring PTH, 25D, Ca and P, and other metabolic parameters back into balance [14].

The available vitamin D therapies in United States (US) for SHPT in adults with stage 3 or 4 CKD and VDI are active vitamin D hormones and analogs (AVD; calcitriol, paricalcitol and doxercalciferol), nutritional vitamin D (NVD: cholecalciferol and ergocalciferol), and extended-release calcifediol (ERC). Concerns regarding the safety of AVD and the clinical effectiveness of NVDs have resulted in major unmet medical needs [4]. The demonstrated ability of ERC to improve vitamin D status and lower elevated PTH in a safe and physiological way presents an attractive alternative [15].

In clinical trials, the available therapeutic options have shown varying efficacy for raising 25D and reducing elevated PTH levels. Randomized clinical trials (RCTs) have shown NVD to be inferior to AVD in controlling rising PTH levels and recent meta-analyses support the conclusion that NVD as first line therapy often delays the introduction of treatments that can provide more effective PTH control [16–20]. Unfortunately, AVDs are associated with increased levels of serum Ca and P and require frequent monitoring for potential hypercalcemia [4, 21] or hyperphosphatemia. The updated KDIGO clinical practice guideline for CKD-mineral and bone disorder (MBD) discourages routine use of AVD in patients with stage 3–4 CKD [4]. AVD therapy may also exacerbate 25D insufficiency via upregulation of CYP24A1, the vitamin D catabolic enzyme [22].

ERC has been evaluated in multiple phase 1 and phase 2 studies, two randomized controlled phase 3 studies and one open-label extension of the phase 3 studies [23]. These clinical trials have established the efficacy of ERC for increasing serum 25D levels and reducing PTH with minimal changes in serum Ca or P levels in adult patients with stage 3–4 CKD and VDI. Corresponding real-world clinical data have confirmed the data obtained from RCTs [24] but real-world comparative data are lacking. The lack of such comparative data led to the current evaluation of real-world clinical experience with the available treatment options to raise serum 25D and reduce elevated PTH during a 12-month period.

## Materials and methods

Medical charts of eligible patients from 15 geographically diverse US nephrology clinics were abstracted for key study variables including patient demographics, medication usage, and serial 25D, PTH, Ca, and P determinations. This retrospective chart review included records from November 30, 2016 through October 11, 2019 and proceeded under an informed consent waiver approved by the Western Institutional Review Board.

**Fig. 1:**
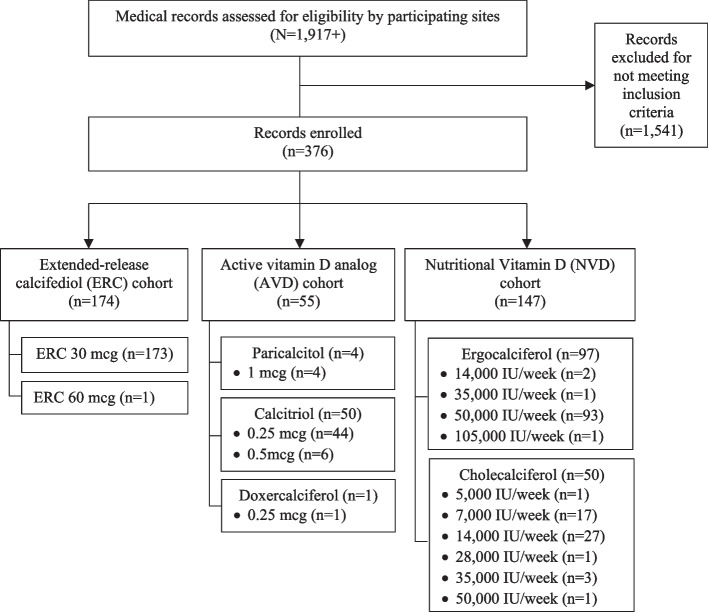
Study CONSORT diagram

Patients eligible for study inclusion were required to have a diagnosis of stage 3 or 4 CKD, defined as an estimated glomerular filtration rate (eGFR) > 15 and < 60 mL/min/1.73m^2^, and a history of VDI and SHPT prior to the index date, defined as the date for initiating the therapy of interest (ERC, AVD or NVD). Only patients with medical records available for at least 6 months prior to and after the index date were included. Eligible patients received the therapy of interest for at least 1 month after initiation and needed to be naïve to ERC and AVD for at least 3 months prior to the index date. In total, over 1917 patients were screened for eligibility. Of these, 1541 were excluded because they did not meet the study inclusion criteria. Enrolled subjects (*n* = 376) were placed into three cohorts (Fig. 1) based on the administered therapy: ERC (*n =* 174), AVD (*n =* 55) and NVD (*n =* 147).

Descriptive analyses were conducted on the data collected. Mean value, standard deviation, and standard errors were calculated for continuous variables. Counts and percentages were computed for any categorical data. Statistical analyses, including t-tests and ANOVA,were used to determine whether there were statistically significant changes to outcomes.

## Results

Of the 376 enrolled patients, 174 (46.3%) initiated treatment with ERC (99.4% at a 30 mcg daily dose), 55 (14.6%) initiated treatment with AVD (80% received calcitriol at 0.25 mcg/ day, 11% received calcitriol at 0.50 mcg/day, 7% received doxercalciferol at 2.5 mcg/day and 2% received paricalcitol at 1.0 mcg/day) and 147 (39.1%) initiated treatment with NVD [weekly oral ergocalciferol (*n* = 97) or cholecalciferol (*n* = 50) at doses of ≥50,000 IU (64.7%), 14,000 to < 50,000 IU (23.1%) or 5000 to < 14,000 IU (12.2%) for ≥7 months (55.8%), 4–6 months (19.0%) or 1–3 months (25.2%)]. The mean (SD) age of the enrolled patients was 69.5 (13.2) years, mean body mass index (BMI) was 32.8(15.2) kg/m^2^, 50.8% were female, 88.8% non-Hispanic and 64.6% Caucasian. BMI was highest among the ERC cohort. ERC and AVD cohorts consisted of more CKD stage 4 patients than stage 3, while the reverse was true for the NVD cohort. Patient demographics and baseline characteristics for all three cohorts are presented in Table 1.

**Table 1:** Patient demographics and baseline characteristics

Variable	Full (*n =* 376)	ERC (*n =* 174)	AVD (*n =* 55)	NVD (*n =* 147)
Age, Mean (SD; yrs)	69.5 (13.2)	69.0 (13.2)	71.8 (13.1)	69.3 (13.4)
Male, n (%)	185 (49.2%)	84 (48.3%)	30 (54.5%)	71 (48.3%)
Hispanic, n (%)	42 (11.2%)	27 (15.5%)	4 (7.3%)	11 (7.5%)
Race, n (%)				
Caucasian	243 (64.6%)	113 (64.9%)	35 (63.6%)	95 (64.6%)
African American	75 (19.9%)	34 (19.5%)	10 (18.2%)	31 (21.1%)
Asian American	1 (0.3%)	0 (0%)	0 (0%)	1 (0.7%)
Native American	1 (0.3%)	0 (0%)	1 (1.8%)	0 (0%)
Other	27 (7.2%)	19 (10.9%)	2 (3.6%)	6 (4.1%)
Not Available	29 (7.7%)	8 (4.6%)	7 (12.7%)	14 (9.5%)
BMI, Mean (SD; kg/m^2^)	32.8 (15.2)	34.2 (20.7)	29.4 (7.2)	32.4 (7.6)
Primary Insurance Status, n (%)				
Commercial	104 (27.7%)	47 (27.0%)	16 (29.1%)	41 (27.9%)
Medicare	235 (62.5%)	103 (59.2%)	36 (65.5%)	96 (65.3%)
Medicaid	20 (5.3%)	11 (6.3%)	3 (5.5%)	6 (4.1%)
Tricare/Other Military or VA	4 (1.1%)	2 (1.1%)	0 (0.0%)	2 (1.4%)
Uninsured	1 (0.3%)	1 (0.6%)	0 (0.0%)	0 (0.0%)
Other	8 (2.1%)	7 (4.0%)	0 (0.0%)	1 (0.7%)
Unknown	4 (1.1%)	3 (1.7%)	0 (0.0%)	1 (0.7%)
Primary Cause of CKD, n^a^ (%)				
Known cause	*n =* 113	*n =* 69	*n =* 13	*n =* 31
Diabetes	44 (38.9%)	30 (43.5%)	2 (15.4%)	12 (38.7%)
Hypertension	64 (55.6%)	36 (52.2%)	11 (84.6%)	17 (54.8%)
Other	5 (4.4%)	3 (4.3%)	0 (0.0%)	2 (6.5%)
CKD Stage, n (%)				
CKD Stage 3	204 (54.3%)	81 (46.6%)	21 (38.2%)	102 (69.4%)
CKD Stage 4	172 (45.7%)	93 (53.4%)	34 (61.8%)	45 (30.6%)
Comorbidities, n (%)				
Diabetes	194 (51.6%)	90 (51.7%)	29 (52.7%)	75 (51.0%)
Hypertension	303 (80.6%)	128 (73.6%)	46 (83.6%)	129 (87.8%)
Anemia	151 (40.2%)	67 (38.5%)	23 (41.8%)	61 (41.5%)
Heart Failure	48 (12.6%)	14 (8.0%)	10 (18.2%)	24 (16.3%)
Coronary artery disease	42 (11.2%)	17 (9.8%)	5 (9.1%)	20 (13.6%)
Angina	3 (0.8%)	1 (0.6%)	1 (1.8%)	1 (0.7%)
Peripheral vascular disease	7 (1.9%)	3 (1.7%)	1 (1.8%)	3 (2.0%)
Cerebral vascular disease	4 (1.1%)	3 (1.7%)	1 (1.8%)	0 (0.0%)
Cancer	4 (1.1%)	3 (1.7%)	0 (0.0%)	1 (0.7%)
Hyperlipidemia	132 (35.1%)	48 (27.6%)	16 (29.1%)	68 (46.3%)
None	50 (13.3%)	39 (22.4%)	4 (7.3%)	7 (4.8%)
Concomitant Medications, n (%)				
Phosphate Binders	14 (3.7%)	6 (3.4%)	3 (5.5%)	5 (3.4%)
Anemia Medications	71 (18.9%)	25 (14.4%)	15 (27.3%)	31 (21.1%)

*ERC* extended-release calcifediol; *AVD* active vitamin D analog; *NVD* nutritional vitamin D; *BMI* body mass index; *CKD* chronic kidney disease

^a^among those with a known cause

Evaluated treatment characteristics included length of prescription and dose titrations, as well as reasons for dose titrations. Most prescriptions had durations of more than 6 months (72.2%): the mean (SD) observed prescription length of ERC was 63.5 (36.5) weeks, 51.3 (33.6) weeks for AVD, and 41.5 (32.4) weeks for NVD. A few patients (1.7%) up-titrated dose in the ERC cohort (from 30 to 60 mcg/day) but patients in the AVD and NVD cohorts maintained a constant dose throughout the study.

In the ERC cohort, the baseline 25D and PTH levels averaged 20.3 ± 0.7 (SE) ng/mL and 181.4 ± 7.4 pg/mL, respectively. ERC treatment raised 25D by 23.7 ± 1.6 ng/mL (*p* < 0.001) and decreased PTH by 34.1 ± 6.6 pg/mL or 18.8% (p < 0.001) without significant impact on serum Ca and P levels. Serum total alkaline phosphatase (ALP) trended downwards. Additionally, eGFR decreased 3.1 ± 0.7 mL/min/1.73m^2^ (p < 0.001). Mean follow-up times for these laboratory parameters ranged from 23.4 to 28.8 weeks (Table 2). Normalized for duration of the follow-up (mean 28.1 weeks), the mean eGFR decrease per patient-week was 0.11 mL/min/1.73m^2^.

**Table 2:** Primary Analysis – Key Lab Values

	ERC, (*n =* 174)					AVD, (*n =* 55)					NVD, (*n =* 147)				
Lab Value	Pre	Post	∆	Mean_FU_	Med_FU_	Pre	Post	∆	Mean_FU_	Med_FU_	Pre	Post	∆	Mean_FU_	Med_FU_
25D, ng/mL* Mean (SE)	20.3^a^ (0.7)	44.0 (1.7)	23.7^**aa,bb,cc**^ (1.6)	24.6	19.9	23.5 (1.0)	29.0 (1.2)	5.5^**cc**^ (1.3)	21.3	18.1	18.8^aa^ (0.6)	28.5 (1.6)	9.7^**cc**^ (1.5)	21.1	17.6
PTH, pg/mL* Mean (SE)	181.4^bb^ (7.4)	147.4 (7.1)	−34.1^bb,**cc**^ (6.6)	23.4	18.8	156.9 (9.7)	149.9 (11.1)	−7.0 (9.8)	21.6	18.1	134.8 (6.8)	144.4 (9.5)	9.6 (6.0)	20.7	17.4
Ca, mg/dL Mean (SE)	9.2^b^ (0.1)	9.3 (0.1)	0.1 (0.1)	27.8	22.2	9.1 (0.1)	9.3 (0.1)	0.2^**cc**^ (0.1)	21.4	18.1	9.3 (0.1)	9.3 (0.1)	0.0 (0.1)	20.4	16.1
P, mg/dL Mean (SE)	3.8 (0.1)	3.9 (0.1)	0.1 (0.1)	28.8	22.1	3.8 (0.2)	3.9 (0.2)	0.1 (0.1)	24.5	18.6	3.7 (0.1)	3.8 (0.1)	0.1 (0.1)	20.5	15.9
ALP, IU/L Mean (SE)	96.9 (3.6)	95.3 (3.8)	−2.1 (1.9)	28.4	21.6	82.1 (7.4)	79.8 (6.5)	−2.3 (2.7)	21.3	18.6	97.0 (3.4)	99.8 (4.0)	2.7 (2.4)	18.3	14.7
eGFR Mean (SE)	31.1^b^ (1.1)	28.0 (0.9)	**−**3.1^**cc**^ (0.7)	28.1	22.2	30.3^b^ (1.4)	28.7 (1.6)	−1.6^**c**^ (0.6)	21.4	18.1	35.7 (1.0)	34.5 (1.0)	−1.2^**c**^ (0.6)	20.0	16.0

^a^Significantly different from AVD, one-way ANOVA with Tukey’s multiple comparision test, *p <* 0.05

^aa^Significantly different from AVD, one-way ANOVA with Tukey’s multiple comparision test, *p <* 0.001

^b^Significantly different from NVD, one-way ANOVA with Tukey’s multiple comparision test, *p <* 0.05

^bb^Significantly different from NVD, one-way ANOVA with Tukey’s multiple comparision test, *p <* 0.001

^c^Significantly different from baseline, *p <* 0.05

^cc^Significantly different from baseline, *p <* 0.001

*ERC* Extended-Release Calcifediol; *AVD* Active Vitamin D; *NVD* Nutritional Vitamin D; 25D, 25-hydroxyvitamin D; *PTH* parathyroid hormone; *Ca* serum calcium; *P* serum phosphorus; *ALP* alkaline phosphatase; *IU* International Units; *eGFR* estimated glomerular filtration rate; *Pre* pretreatment value; *Post* post-treatment value; Δ, Change from Pre to Post-treatment; *Mean*_*FU*_ Mean Follow-up time (weeks); Median_FU_, Median Follow-up time (weeks)

In the AVD cohort, baseline 25D and PTH levels averaged 23.5 ± 1.0 (SE) ng/mL and 156.9 ± 9.7 pg/mL, respectively. Serum 25D rose by 5.5 ± 1.3 ng/mL (*P* < 0.001) without statistically significant impact on PTH and serum P levels. Serum total ALP trended downwards. Additionally, serum Ca levels elevated by 0.2 ± 0.1 mg/dL (*p* < 0.001) and eGFR decreased by 1.6 ± 0.6 mL/min/1.73m^2^ (*p* < 0.01). Mean follow-up times ranged from 21.3 to 24.5 weeks (Table 2). Normalized for duration of the follow-up (mean 21.4 weeks), the mean eGFR decrease per patient-week was 0.08 mL/min/1.73m^2^.

In the NVD cohort, baseline 25D and PTH levels averaged 18.8 ± 0.6 (SE) ng/mL and 134.8 ± 6.8 pg/mL, respectively. Serum 25D increased by 9.7 ± 1.5 ng/mL (*p* < 0.001) without significant impact on PTH or serum Ca and P levels. Serum total ALP trended upwards. Additionally, eGFR decreased 1.2 ± 0.6 mL/min/1.73m^2^ (*p* < 0.05). Mean follow-up times ranged from 18.3 to 21.1 weeks (Table 2). Normalized for duration of the follow-up (mean 20.0 weeks), the mean eGFR per patient-week decrease was 0.07 mL/min/1.73m^2^.

There were no statistically significant differences in the normalized eGRF decline between groups. Some variations in results were identified within subgroups of the ERC, AVD and NVD cohorts. African-Americans experienced less clinical effectiveness compared to non-African Americans. Among patients with diabetes, hypertension, anemia, or hyperlipidemia, those with comorbidities faced worse clinical effectiveness compared to those without. Across all groups, patients below the BMI threshold of 30 for obesity saw more clinical effectiveness than those above, though ERC was still the most effective of the three treatments for those with higher BMI values.

**Fig. 2:**
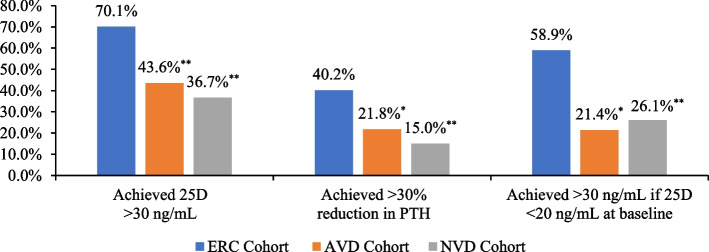
Primary Analysis – Key Endpoints

Additional analyses were conducted to determine effectiveness of therapies based on main parameters of interest (Fig. 2). Within the ERC cohort, 70.1% (*n* = 122) of patients achieved 25D levels of ≥30 ng/mL at follow-up, compared to 43.6% (*n* = 24) and 36.7% (*n* = 54) in the AVD and NVD cohorts, respectively (*p* < 0.001 compared to ERC). Among ERC-treated patients that started at a baseline 25D level of < 20 ng/mL, 58.9% (*n* = 53) of patients achieved a 25D level of ≥30 n/mL by follow-up, compared to 21.4% (*n* = 3) and 26.1% (*n* = 23) in the AVD and NVD cohorts, respectively (*p* < 0.05 or 0.001 compared to ERC). In regard to achievement of a ≥ 30% reduction in PTH over the duration of the study, 40.2% (*n* = 70) of ERC-treated patients achieved this endpoint, compared to 21.8% (*n* = 12) and 15.0% (*n* = 22) in the AVD and NVD cohorts, respectively (*p* < 0.05 or 0.001 compared to ERC).

In addition, the ERC cohort had a much greater percentage of patients achieving 25D levels of at least 50 ng/mL than the AVD and NVD cohorts (51.6, 12.5, and 18.5%, respectively), which may help explain the observed differences in PTH reduction between cohorts.

## Discussion

In the real-world clinical setting, ERC was found to be the only treatment which significantly reduced mean PTH in US pre-dialysis CKD patients. The lack of PTH lowering with AVD was surprising and possibly due to routine prescription of insufficient dosages without up-titration, but was expected with NVD, as numerous meta-analyses [4, 16–20] have concluded that PTH lowering with cholecalciferol or ergocalciferol supplements is unproven and clinically insignificant. NVD has been shown to be less effective in raising serum 25D in overweight patients [25, 26] and most CKD patients are overweight, developing kidney disease from hypertension or type 2 diabetes, both common complicaitons of obesity. Disparities in PTH-lowering efficacy in this study were not related to differences in the bioavailabilities of the various treatments, as ERC has a slow-release formulation with only 25% bioavailabilty [23] compared to substantially higher bioavailabities reported for AVD and NVD [27, 28].

Patients treated with ERC also demonstrated the largest mean increase in serum 25D, which was significantly greater than mean increases observed with AVD and NVD (23.7 vs. 5.5 and 21.1 ng/mL for AVD and NVD). ERC was the only therapy which raised mean 25D to ≥30, and patients treated with ERC achieved more frequently 25D > 30 ng/mL and > 30% reduction in PTH in the follow-up period. Elevations of 25D levels of at least 50 ng/mL have been reported to be required for ≥30% reductions of PTH in CKD stage 3 and 4 patients [29]. ERC raises serum 25D gradually due to its formulation which releases calcifediol over a 12-hour period and, as a result, has been shown in RCTs to safely raise 25D to well over 50 ng/mL when administered in appropriately high dosages (30 or 60 mcg/day) irrespective of a patient’s body weight [26]. Calcifediol differs from NVD in that it is more bioavailable, requires no hepatic activation, is more water soluble, and avidly binds the vitamin D binding protein in serum, which together improve its effectiveness in raising serum 25D [26]. The nominal rise in mean 25D with AVD was likely a result of unauthorized vitamin D supplementation, as AVD is known to increase the expression of CYP24A1 which, in the absence of supplementation, would reduce 25D levels [22].

The more favorable clinical effectiveness results among ERC-treated patients were accomplished when virtually all received a 30 mcg/day dose rather than being force-titrated up to 60 mcg/day per previous RCTs [23]. More frequent up-titration up to 60 mcg/day would have further improved ERC clinical effectiveness results.

Clinical effectiveness with ERC in this real-world study was unaccompanied by any safety concerns. ERC administration had no significant impact on the key safety outcomes of serum Ca and P levels, while AVD administration produced a small, but significant, increase in serum Ca levels.

Subgroup analyses among the treatment cohorts suggested that some patient characteristics may have impacted treatment clinical effectiveness results. Specifically, African-American patients and those possessing certain comorbidities, such as diabetes, hypertension, anemia, or hyperlipidemia, experienced less clinical effectiveness with treatment, whereas patients remaining below the BMI threshold of 30 for obesity saw greater clinical effectiveness. When the analyses were stratified by CKD stage, ERC consistently had higher mean increases in 25D levels and greater reductions on PTH than found in the AVD and NVD cohorts. Due to relatively small sample sizes among the subgroups analyzed by treatment cohort, no strong conclusions can be made. However, the results do point to the potential need for more monitoring of these populations not experiencing as positive clinical outcomes as others and the importance of further research into what characteristics or factors are driving clinical outcomes and how they can be addressed or mitigated to improve clinical effectiveness.

## Limitations

This study was not powered or of sufficient duration to examine endpoints related to cardiovascular disease, fractures, hospitalization rates and mortality. The study merely aimed to analyse the effects of the three therapeutic options on serum total 25D, plasma PTH and serum ALP, Ca and P, all of which when abnormal are associated with unwanted outcomes in patients with CKD [4]. The nature of the study design, as with any retrospective chart review, led to certain limitations: medical records may have been incomplete, due to missing data points, or involvement of multiple sites during a patient’s care. To highlight the point of incomplete charts/ missing data, we set out to capture data on proteinurea, as proteinuria is one of the most important surrogate marker of progression of kidney disease but also adverse CV outcomes. Unfortunately we were unsuccessful as most facilities are did not have data for the observation period or there were too many variations, for example, some ordered protein to creatinine ratio, some ordered urine for albumin to creatine ratio, ad some ordered only urine for protein or urine for albumin without urine creatinine, making it impossible to meaningfully analyze the data. Selection bias towards a more severe population may have been introduced by the inclusion criterion ensuring patients had documentation of serum 25D and PTH levels before and after the index date. Differences in laboratory methodologies between study sites may have introduced bias into serum 25D and PTH changes. Also, cohort results should be interpreted in the context of duration of follow-up times as there was some variation between cohorts.

## Conclusions

Results from the current study highlight ERC’s strong potential to successfully address unmet treatment needs associated with AVD and NVD in patients with SHPT, stage 3–4 CKD and VDI. These real-world data demonstrated ERC’s ability to reliably increase serum 25D and reduce elevated PTH levels without significant negative clinical impact on serum Ca and P levels. Future research into factors influencing clinician patient follow-up and dose titration practices, as well as what patient-related characteristics are influential in treatment outcomes, can further contribute toward informing optimal SHPT management and treatment practices to improve clinical effectiveness and safety.

## Data Availability

The datasets used and/or analyzed during the current study are available from the corresponding author on reasonable request.

## Abbreviations

1,25 D: 1,25-dihydroxyvitamin D
25D: 25-hydroxyvitamin D
ALP: Alkaline phosphatase
AVD: Active vitamin D analog
BMI: Body mass index
Ca: Calcium
CKD: Chronic kidney disease
ERC: Extended-release calcifediol
IU: International Units
NVD: Nutritional vitamin D
PTH: Parathyroid hormone
P: Phosphorus
SHPT: Secondary hyperparathyroidism
VDI: Vitamin D insufficiency
